# S-Allyl Cysteine Alleviates Hydrogen Peroxide Induced Oxidative Injury and Apoptosis through Upregulation of Akt/Nrf-2/HO-1 Signaling Pathway in HepG2 Cells

**DOI:** 10.1155/2018/3169431

**Published:** 2018-11-01

**Authors:** Chitra Basu, Runa Sur

**Affiliations:** Department of Biophysics, Molecular Biology and Bioinformatics, University of Calcutta, 92 A. P. C. Road, Kolkata 700009, India

## Abstract

Hydrogen peroxide (H_2_O_2_) mediated oxidative stress leading to hepatocyte apoptosis plays a pivotal role in the pathophysiology of several chronic liver diseases. This study demonstrates that S-allyl cysteine (SAC) renders cytoprotective effects on H_2_O_2_ induced oxidative damage and apoptosis in HepG2 cells. Cell viability assay showed that SAC protected HepG2 cells from H_2_O_2_ induced cytotoxicity. Further, SAC treatment dose dependently inhibited H_2_O_2_ induced apoptosis via decreasing the Bax/Bcl-2 ratio, restoring mitochondrial membrane potential (∆Ψ_m_), inhibiting mitochondrial cytochrome c release, and inhibiting proteolytic cleavage of caspase-3. SAC protected cells from H_2_O_2_ induced oxidative damage by inhibiting reactive oxygen species accumulation and lipid peroxidation. The mechanism underlying the antiapoptotic and antioxidative role of SAC is the induction of the heme oxygenase-1 (HO-1) gene in an NF-E2-related factor-2 (Nrf-2) and Akt dependent manner. Specifically SAC was found to induce the phosphorylation of Akt and enhance the nuclear localization of Nrf-2 in cells. Our results were further confirmed by specific HO-1 gene knockdown studies which clearly demonstrated that HO-1 induction indeed played a key role in SAC mediated inhibition of apoptosis and ROS production in HepG2 cells, thus suggesting a hepatoprotective role of SAC in combating oxidative stress mediated liver diseases.

## 1. Introduction

Oxidative stress in liver hepatocytes underlies various liver diseases [1]. Hydrogen peroxide (H_2_O_2_) plays a major role in inducing liver oxidative stress, by disrupting the cellular redox circuitry that depends on the redox state of various signaling molecules behaving as redox sensitive molecular switches, or by directly damaging cellular macromolecules including DNA, proteins, and lipids. This alters many fundamental cellular functions including proliferation, differentiation, migration and adhesion [1] and eventually results in sustained hepatocyte apoptosis, a pathological condition frequently associated with the progression of several liver diseases such as hepatic ischemia-reperfusion (I/R) injury, alcoholic liver disease, nonalcoholic fatty liver disease, and hepatitis [2, 3].

H_2_O_2_ levels that induce oxidative stress have been shown to downregulate heme oxygenase-1 (HO-1), a phase II anti-oxidant enzyme, involved in the rate limiting step of heme metabolism that catalyzes the conversion of heme into carbon monoxide and biliverdin. Several studies have depicted that, induction of HO-1 expression interferes with the progression of a number of hepatic pathophysiological conditions including ischemia/ reperfusion (I/R) injury, liver inflammation, hepatic fibrosis and hepatitis [4]. It has also been shown that HO-1 plays a role in cellular defense mechanism against oxidative stress induced apoptotic cell death [5–7].

S-allyl cysteine (SAC), a potential antioxidant found in the aged garlic extract (AGE) [8], has been reported to possess cytoprotective effects [9]. SAC has many advantages over other garlic compounds owing to the facts that SAC is odourless and less toxic, pharmacokinetic studies show that it has 98 percent bioavailability [10], it is the only reliable marker used for studies involving oral garlic intake because it is detectable and increases quantitatively in the blood and it is the only constituent of garlic that does not induce P450 isozymes in the body suggesting that SAC will not cause P450-induced contraindications with drugs [10].

Several* in vivo* studies have suggested SAC to protect from oxidative stress induced liver injury. SAC has shown efficacy in protecting from carbon tetrachloride induced liver cirrhosis [11] and liver injury [9]. SAC improved nonalcoholic fatty liver disease in rats with type 2 diabetes via regulation of hepatic lipogenesis and glucose metabolism [12]. SAC alleviated chromium (VI)-induced hepatotoxicity in rats by inhibiting inflammatory markers [13]. However the detailed mechanism behind the antioxidative and antiapoptotic effects of SAC has not been elucidated. The present study has been designed to investigate the mechanism behind the anti-oxidative and anti-apoptotic potential of SAC in hydrogen peroxide stimulated HepG2 cells, a widely used* in vitro* model for the study of oxidative injury in liver. For the first time we demonstrate in our study that SAC alleviates hydrogen peroxide induced oxidative injury and apoptosis through upregulation of Akt/Nrf-2/HO-1 signaling pathway in HepG2 cells.

## 2. Materials and Methods

### 2.1. Materials

S-allyl cysteine was purchased from Abcam. Trypan blue, 2′,7′-dichlorodihydrofluorescein diacetate (DCFH_2_-DA),5,5′,6,6′-tetrachloro-1,1′,3,3′-tetraethyl-imidacarbocyanine iodide (JC-1), and Wortmannin were purchased from Sigma-Aldrich, USA. Dulbecco's modified eagle medium (DMEM), fetal bovine serum (FBS), penicillin-streptomycin, and trypsin-EDTA solutions were purchased from HiMedia, India. INTERFERin siRNA transfection reagent was purchased from Polyplus, USA. Taq polymerase and dNTPs were purchased from Thermo Fisher Scientific, USA. Random hexamer primer and RiboLock RNase inhibitor, and RevertAid reverse transcriptase were purchased from Thermo Scientific, USA. Anti-*α*-tubulin antibody was purchased from BioBharati LifeScience Pvt. Ltd., India. Anti-Nrf-2, anti-Akt, anti-p-Akt, Bcl2, Bax, caspase 3, and cytochrome c antibodies were purchased from R&D systems, USA. Primers for real time PCR were purchased from Integrated DNA Technologies, USA.

### 2.2. Cell Culture

HepG2 cells were obtained from National Centre for Cell Science (NCCS), Pune, India, and were cultured in DMEM medium supplemented with 10 % FBS and 1% penicillin-streptomycin at 37°C with 5% CO_2_.

### 2.3. Cell Viability Assay

Cell viability was measured using trypan blue exclusion method [14]. Briefly, HepG2 cells were seeded in 24-well plates at a density of 1×10^5^ cells per well for 24 h. Then, dose response and time course experiments were performed with H_2_O_2_. Additionally, cells were either left untreated or pre-treated with different concentrations of SAC in the presence or absence of H_2_O_2_ for 24 h. Also, cells were either left untreated or treated with H_2_O_2_ followed by treatment with SAC (150 *μ*M) for different time points (posttreatment). Then the cells were harvested and trypan blue solution (0.4 %) was added to the cell suspensions in a ratio of 1:1. Finally the numbers of total and dead cells were counted using hemocytometer. The percentage of viable cells was calculated and plotted.

### 2.4. Cell Cycle Analysis by Flow Cytometry

Cell cycle analysis was performed according to a previously described method [15]. Briefly 4 × 10^5^ cells were seeded in each well of a 6-well plate and incubated for 24 h. Then cells were either left untreated or treated with SAC for 2 h followed by exposure to 1 mM H_2_O_2_ for 24 h. Harvested cells were then subjected to chilled 70 % ethanol fixation and RNase A (100 *μ*g/ml) treatment followed by treatment with propidium iodide (120 *μ*g/ml) to stain cellular DNA. The percentages of cells in different phases of the cell cycle were analysed using FACS Verse (Becton–Dickinson) equipped with 405 nm (Violet), 488 nm (Blue) and 640 nm (Red) solid state laser light by acquiring at least 10,000 cells per sample. Data were analyzed using FACSuite software [Version 1.0.3.2942].

### 2.5. Determination of Apoptosis

Apoptotic cells were detected by Annexin V/PI double staining method. Initially, each well of a 6-well plate was seeded with 4×10^5^ cells and incubated for 24 h. Then cells were either left untreated or pretreated with SAC for 2 h followed by exposure to 1 mM H_2_O_2_ in the presence of SAC for 24 h. Cells were subjected to Annexin-V and PI staining with Annexin-V-FLUOS Staining Kit (Roche Diagnostics) according to the manufacturer's protocol. Finally, the distribution of the apoptotic cells was measured using FACS Verse (Becton Dickinson). The values are represented as fold change over control.

### 2.6. Measurement of Mitochondrial Membrane Potential

Mitochondrial membrane potential (∆Ψ_m_) was measured using the cationic dye JC-1 that accumulates in mitochondria. Initially, each well of a 6-well plate was seeded with 4×10^5^ cells and incubated for 24 h. Then cells were either left untreated or pretreated with SAC for 2 h followed by exposure to 1 mM H_2_O_2_ in the presence of SAC for 6 h. Thereafter the cells were washed with PBS followed by staining with 20 *μ*M of JC-1 for 30 min at 37°C. Cells were washed with phosphate buffer saline followed by analysis in FACS Verse. At high concentration (indicative of high ∆Ψ_m_) JC-1 accumulates in the intact mitochondria as aggregates yielding red fluorescence (590 nm). However, at low concentration (indicative of low ∆Ψ_m_) JC-1 remains predominantly as monomers in the depolarized mitochondria yielding green fluorescence (535 nm) [16].

### 2.7. Measurement of ROS Levels

ROS levels were measured by DCFH_2_-DA staining followed by FACS analysis according to a previously described method [15]. Initially, each well of a 6-well plate was seeded with 4×10^5^ cells and incubated for 24 h. Then cells were either left untreated or pre-treated with SAC for 2 h followed by exposure to 1 mM H_2_O_2_ in the presence of SAC for another 2 h. Additionally, cells were pretreated with 1 mM H_2_O_2_ followed by SAC treatment or none for different time points. Then cells were stained with 20 *μ*M of DCFH_2_-DA in serum free media for 30 min at 37°C. DCF fluorescence, an indicator of ROS levels, was measured using FACS Verse.

### 2.8. Measurement of Lipid Peroxidation

The extent of lipid peroxidation was determined using a previously established protocol [17]. Briefly, HepG2 cells were seeded in 6-well plates at a density of 4×10^5^ and incubated for 24 h. Then cells were either left untreated or pretreated with SAC for 2 h followed by exposure to 1 mM H_2_O_2_ for 6 h to stimulate oxidative injury.

After treatment, cells were harvested and lysed by sonicating for 10 sec. Lipid peroxidation was estimated by thiobarbituric acid-reactive substances (TBARS) method [18] by measuring the levels of malondialdehyde (MDA) produced during cellular lipid peroxidation. In short, the cell lysates were then centrifuged at 10,000* g* for 10 min at 4°C. Then equal volume of TBA solution (0.375 % TBA, 15 % trichloroacetic acid, and 0.25 N HCl) was added to the supernatants and heated for 15 min in a boiling water bath followed by centrifugation at 10,000* g* for 5 min. Finally absorbance of the supernatant was measured at 535 nm. The values are represented as fold change over control.

### 2.9. RNA Extraction, Reverse Transcription and Semiquantitative PCR

Cells were seeded in 6-well plates and after treatment; total RNA was isolated using TRIzol reagent (Invitrogen). Equal amount (1.5 *μ*g) of RNA from different samples was subjected to reverse transcription for cDNA synthesis using random hexamer primers, dNTPs, RNase inhibitor, buffer, and reverse transcriptase enzyme. PCR was performed with cDNA as template and gene specific primers for HO-1 and *β*-actin using Taq DNA polymerase. The primer sequences for human HO-1 gene were 5′-CGGGCCAGCAACAAAGTG-3′ (forward), 5′-AGTGTAAGGACCCATCGGAGAA-3′ (reverse) and primer sequences for human *β*-actin gene were 5′-CTTCCTTCCTGGGCAT-3′ (forward), and 5′-CAGGGTACATGGTGGTG-3′ (reverse). PCR was performed using the following condition: 94°C for 5 min followed by 35 cycles of 94°C for 30 s, 50°C for 30 s, and 72°C for 30 s, followed by 72°C for 7 min. The amplified products were resolved by 8% polyacrylamide gel electrophoresis and stained with ethidium bromide, and photograph was taken under UV light.

### 2.10. Protein Extraction and Western Blot Analysis

Whole cell protein extract was prepared by washing the harvested cells with phosphate buffered saline and resuspending the cell pellet in RIPA lysis buffer (50 mM Tris-HCl pH 7.4, 150 mM NaCl, 0.1 % SDS, 1 % NP40, 0.5 % sodium deoxycholate, 0.2 mM Na_3_VO_4_, 50 mM NaF, 2 mM EDTA, and protease inhibitor cocktail) for 20 min on ice followed by centrifugation at 13,000* g* for 15 min at 4°C. The supernatant was used as whole cell lysate. Cytosolic and nuclear extracts were prepared using a previously described method [19] with minor modifications. Cells were washed with phosphate buffered saline and centrifuged at 400* g* for 10 min at 4°C. The pellet was resuspended in 300 *μ*l cytosolic extraction buffer (10 mM HEPES pH 7.9, 10 mM KCl, 1 mM EDTA, 1 mM EGTA, 1mM DTT, 0.5 mM PMSF, and 0.5 % NP40) containing protease inhibitor cocktail and incubated on ice for 15 min. After rapid mixing for 15 sec followed by centrifugation at 4000* g* for 1 min at 4°C, the nuclei were pelleted and the supernatant containing the cytosolic extract was collected. The pellet containing the nuclei was resuspended in 100 *μ*l nuclear extraction buffer (10 mM HEPES pH 7.9, 0.4 M KCl, 1 mM EDTA, 1 mM EGTA, 1mM DTT, 0.5 mM PMSF, 0.5 % NP40, and 20 % glycerol) and vortexed followed by centrifugation at 13,000* g* for 15 min at 4°C. The supernatant contained the nuclear extract.

For detection of cytochrome c protein released in the cytosol, cytosolic extract was prepared using a previous method [20] with few modifications. Briefly cells were washed with phosphate buffered saline and homogenized using homogenization buffer (10 mM HEPES pH 7.4, 250 mM sucrose, 10 mM KCl, 1.5 mM MgCl_2_, 1 mM EDTA, 1 mM EGTA, 1 mM DTT, and protease inhibitor cocktail) followed by centrifugation at 2000* g* for 5 min at 4°C. The supernatant was centrifuged at 13000* g* for 25 min at 4°C. The supernatant was used as the cytosolic extract.

Protein concentration in the cell lysates was determined using Lowry's assay. Equal amount of protein was loaded and separated on SDS-PAGE and transferred to PVDF membrane. The membrane was blocked with 5 % BSA followed by hybridization with respective primary and secondary antibodies. Protein bands were visualized using Luminol reagent (Sigma) and exposing the blot to X-ray film. The image was visualized using gel documentation system (Bio-Rad) and densitometry analysis was performed using Quantity One software. The intensity of protein bands was normalized to their respective internal control.

### 2.11. Small Interfering RNA (siRNA) Transfection

Predesigned small interfering RNA (siRNA) against HO-1 (sense strand CAGUUGCUGUAGGGCUUUAdTdT) and control siRNA (SR-CL000-005) were purchased from Eurogentec, Belgium. Transient transfection of the siRNA was performed using the INTERFERin transfection reagent according to the manufacturers' protocol. siRNA transfection was carried out for 72 h.

### 2.12. Statistical Analysis

All data are expressed as mean ± SD and are representative of at least three independent experiments. P value of < 0.05 was considered significant. Student's t test was used to assess differences between groups. IC_50_ value of H_2_O_2_ was calculated using KyPlot version 2.0 beta 15 (32 bit).

## 3. Results

### 3.1. SAC Inhibits H_2_O_2_-Induced Cytotoxicity in HepG2 Cells

**Figure 1:**
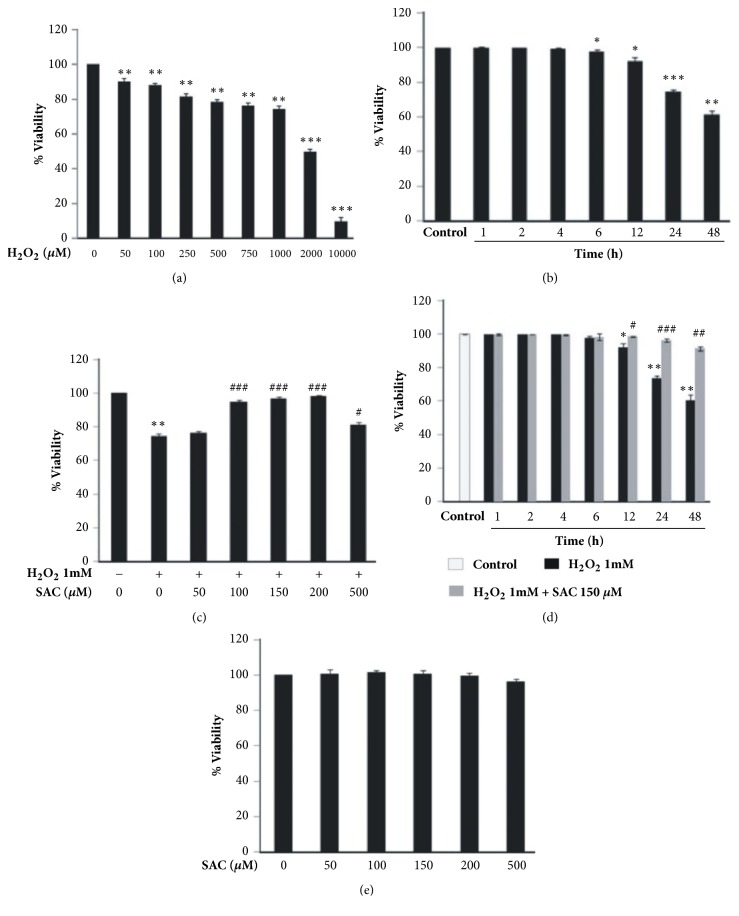
SAC inhibits H_2_O_2_ induced cytotoxicity in HepG2 cells. Cytotoxicity was measured by trypan blue exclusion method. (a) Cells were treated with different concentrations of H_2_O_2_ for 24 h. (b) Cells were treated with 1 mM H_2_O_2_ for different time points. (c) Cells were either left untreated or treated with SAC for 2 h followed by treatment with 1 mM H_2_O_2_ and SAC for 24 h. (d) Cells were either left untreated or treated with 1 mM H_2_O_2_ for 1 h followed by treatment with SAC (150 *μ*M) and H_2_O_2_ for the indicated time points. (e) Cells were treated with different concentrations of SAC for 24 h. *∗*P<0.05, *∗∗*P<0.01, and *∗∗∗*P<0.001 compared to the control group. #P<0.05, ##P<0.01, and ###P<0.001 compared to the H_2_O_2_ treated group.

To determine the proper working concentration of H_2_O_2_, trypan blue exclusion method was performed with HepG2 cells treated with different doses of H_2_O_2_. According to Figure 1(a), H_2_O_2_ treatment inhibited HepG2 cell viability in a dose dependent manner. 1 mM cytotoxic dose of H_2_O_2_, a concentration below its IC_50_ value (2 mM), was selected as a standard concentration for subsequent experiments. We also performed a time course experiment with 1 mM H_2_O_2_ and found that cell viability gradually decreased with increase in incubation time beginning from 6 h (Figure 1(b)).

In order to determine the cytoprotective concentrations of SAC, HepG2 cells were treated with different concentrations of SAC followed by treatment with H_2_O_2_. In the presence of 100, 150 and 200 *μ*M SAC, cell viability increased to 95 ± 1 %, 97 ± 1 % and 98.3 ± 0.6 %, respectively, as compared to the H_2_O_2_ treated group (74.3 ± 1.5%) (Figure 1(c)). Higher concentration of SAC (500 *μ*M) decreased cell viability to 81.3 ± 1.5 % when added with H_2_O_2_ (Figure 1(c)). Therefore, the cytoprotective concentration of SAC to be used for the subsequent experiments was determined to be between 100 and 200 *μ*M. Next we performed a time course experiment in which we pretreated cells with 1 mM H_2_O_2_ for 1 h followed by SAC treatment (150 *μ*M) in the presence of H_2_O_2_ for various time-points (Figure 1(d)). We found that 1 mM H_2_O_2_ treatment induced cell death in a time dependent manner beginning from 6 h and SAC posttreatment reversed this effect. Interestingly we found that SAC dependent cytoprotection was 6.8 % at 12 h, 22.9 % at 24 h and 30.8 % at 48 h when compared to their respective H_2_O_2_ treated control. Thus SAC treatment evoked an increase in cytoprotection compared to control with an increase in time of treatment or an increase in cytotoxicity. To determine whether SAC alone had any effect on cell viability, we treated HepG2 cells with different concentrations of SAC (Figure 1(e)). The tested concentrations of SAC did not affect cell viability up to 200 *μ*M; however low amount of toxicity was observed with 500 *μ*M SAC (value decreased from 100 % to 96 ± 1.8 %).

### 3.2. SAC Inhibits H_2_O_2_-Induced Apoptosis in HepG2 Cells

**Figure 2:**
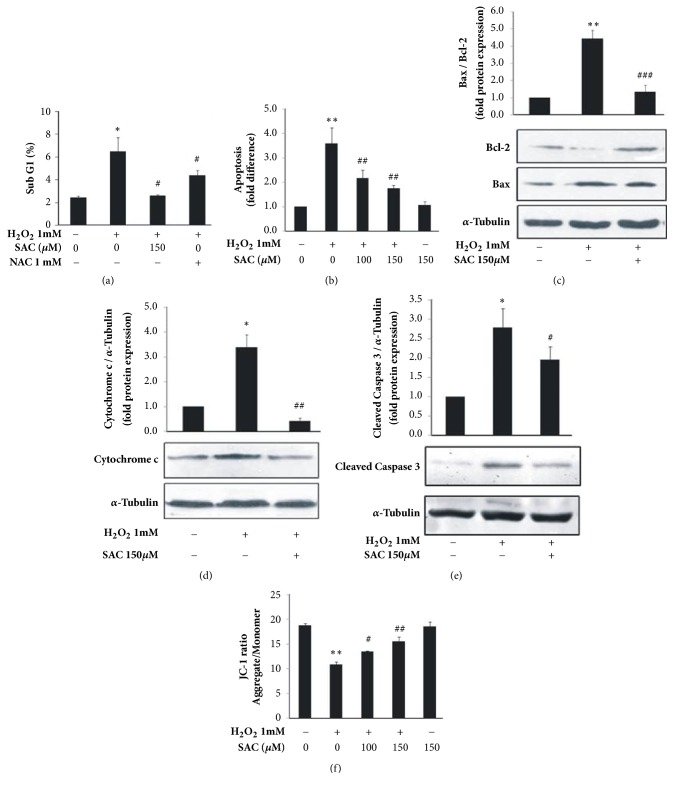
SAC inhibits H_2_O_2_ induced apoptosis in HepG2 cells. Cells were treated with 1 mM H_2_O_2_ for 24 h with or without treatment with SAC. (a) Cell cycle analysis was performed using PI staining followed by FACS analysis. (b) Annexin V/PI double staining was performed followed by FACS analysis. We normalized the apoptosis data with the control and have represented the data in terms of fold change in apoptosis over control. Bar graph represents mean ± SD of four independent experiments. (c) Bax and Bcl-2 protein expression was determined in the whole cell extract by western blot. Bax and Bcl-2 levels were normalized to *α*-Tubulin, an internal loading control. The Bax/Bcl-2 ratio was determined using densitometry and the fold protein expression is represented as a bar graph. (d) Protein level of cytochrome c in the cytosolic extract was determined by western blot. *α*-Tubulin was used as an internal loading control. The cytochrome c/*α*-Tubulin ratio was determined using densitometry and the fold protein expression is represented as a bar graph. (e) Protein level of cleaved caspase 3 was determined by western blot. *α*-Tubulin was used as an internal loading control. The cleaved caspase 3/*α*-Tubulin ratio was determined using densitometry analysis and the fold protein expression is represented as a bar graph. For all c, d, and e densitometry data (bar graph) represents mean ± SD of three independent experiments and a representative blot is shown below. (f) Mitochondrial membrane potential (∆Ψm) was estimated by JC-1 staining followed by FACS analysis. The ratio of aggregate and monomeric form of JC-1 is plotted. *∗*P<0.05 and *∗∗*P<0.01 compared to the control group. #P<0.05, ##P<0.01, and ###P<0.001 compared to the H_2_O_2_ treated group.

During acute and chronic liver injury H_2_O_2_ triggers hepatocyte apoptosis [21]. To investigate whether the cytoprotective role of SAC against H_2_O_2_ induced cytotoxicity in HepG2 cells is mediated via its ability to inhibit apoptosis, cell cycle analysis was performed using propidium iodide (PI) staining followed by flow cytometry.  Figure 2(a) demonstrates that H_2_O_2_ treatment leads to accumulation of 6.5 ± 1.2 % cells in sub-G1 phase as compared to 2.4 % ± 0.1 % cells in the untreated group. However, we found that this H_2_O_2_ induced accumulation of cells in sub-G1 phase decreased (2.6 ± 0.1%) on treatment with 150 *μ*M SAC, indicating that SAC inhibited H_2_O_2_ mediated accumulation in sub-G1 phase. N-acetyl cysteine (NAC) was used as a positive control for the experiment.

Apoptosis specific Annexin V and PI double staining assay (Figure 2(b)) demonstrates that a significant (P<0.01) increase in apoptotic cells was observed in H_2_O_2_ treated HepG2 cells (3.6 ± 0.7 fold) in comparison to untreated cells. However, SAC treatment dose dependently inhibited H_2_O_2_ induced apoptosis and decreased levels to 2.2 ± 0.3 fold for 100 *μ*M and 1.7 ± 0.1 fold for 150 *μ*M of SAC in HepG2 cells. We treated cells with SAC alone (150 *μ*M) and found that SAC showed no significant effect on apoptosis compared to the control (Figure 2(b)).

### 3.3. SAC Inhibits H_2_O_2_ Mediated Increase in Bax/Bcl-2 Ratio, Cytochrome c Release, and Caspase 3 Cleavage in HepG2 Cells

An increase in the ratio of proapoptotic Bax and antiapoptotic Bcl-2 induces mitochondrial cytochrome c release, a hallmark of apoptosis [22]. Release of cytochrome c into the cytosol in turn activates downstream caspases via cleavage, one of which is caspase 3 [23]. We found that H_2_O_2_ treatment upregulated Bax and downregulated Bcl-2 expression in HepG2 cells resulting in the increase of Bax/Bcl-2 ratio (4.4 ± 0.5) as compared to the control (1) (Figure 2(c)) and SAC treatment downregulated Bax and upregulated Bcl-2 thus decreasing the Bax/Bcl-2 ratio (1.3 ± 0.4) in comparison to H_2_O_2_ treated cells.

In addition, we found that H_2_O_2_ treatment increased the cytosolic levels of cytochrome c (3.4 ± 0.5) as compared to the control (1) and SAC treatment decreased these cytosolic levels of cytochrome c (0.4 ± 0.1) (Figure 2(d)). Investigating cleavage of downstream caspase 3 we found that H_2_O_2_ treatment increased levels of cleaved caspase 3 (2.8 ± 0.5) compared to control (1) and SAC treatment decreased caspase 3 cleavage (2 ± 0.3) (Figure 2(e)). These results demonstrate that SAC inhibits H_2_O_2_ induced apoptosis through modulation of the Bcl-2/Bax dependent cell death pathways. *α*-Tubulin was used as an internal control.

### 3.4. SAC Inhibits H_2_O_2_ Mediated Decrease of Mitochondrial Membrane Potential (∆Ψ_m_) in HepG2 Cells

Release of cytochrome c from mitochondria is closely associated with decrease in mitochondrial membrane potential (∆Ψ_m_) [24]. H_2_O_2_ has been reported to decrease ∆Ψ_m_ in HepG2 cells [20]. In order to investigate the effect of SAC on H_2_O_2_ mediated loss of mitochondrial membrane potential (∆Ψ_m_) in HepG2 cells, we performed a well-established JC-1 assay followed by flow cytometry. A lower value of aggregate/monomer ratio denotes loss of mitochondrial membrane potential. As indicated in Figure 2(f), H_2_O_2_ treatment leads to decrease in aggregate/monomer ratio (10.9 ± 0.43) as compared to the control (18.7 ± 0.49). However SAC treatment increased the aggregate/monomer ratio (13.48 ± 0.14 %, 15.49 ± 1 % for 100 *μ*M, and 150 *μ*M SAC, respectively) in a dose dependent manner as compared to H_2_O_2_ treated group. This indicates that SAC restored the H_2_O_2_ induced loss of mitochondrial ∆Ψ_m_ values. SAC alone showed no significant change in mitochondrial membrane potential over control (Figure 2(f)).

### 3.5. SAC Inhibits H_2_O_2_ Induced Reactive Oxygen Species (ROS) Accumulation in HepG2 Cells

**Figure 3:**
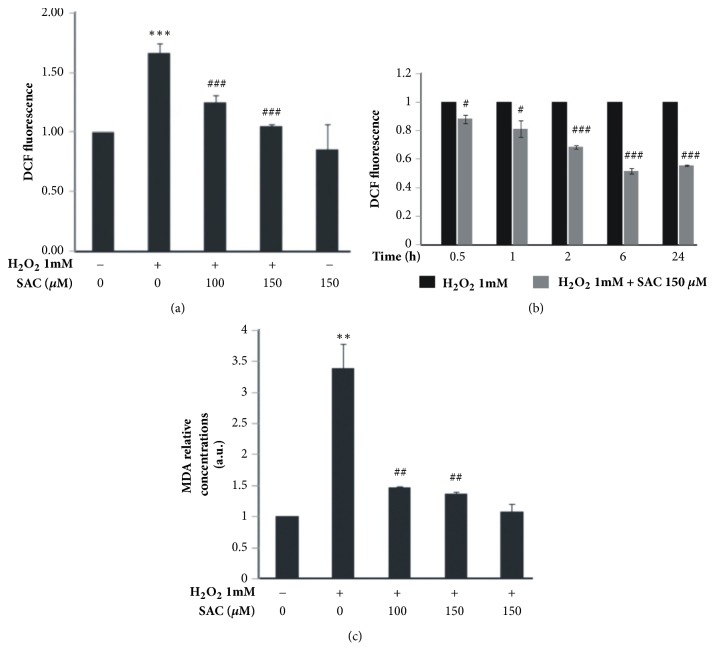
SAC inhibits H_2_O_2_ induced oxidative injury in HepG2 cells. (a) ROS levels were measured by DCFH_2_-DA staining followed by FACS analysis. Cells were either left untreated or treated with SAC for 2 h followed by treatment with 1 mM H_2_O_2_ in the presence of SAC for 2 h. Fluorescence intensities were plotted as fold difference compared to control. (b) Cells were treated with 1 mM H_2_O_2_ for 30 minutes and washed off with PBS and then treated with SAC for different time points. ROS was measured by DCFH_2_-DA staining followed by FACS analysis. Fluorescence intensities were plotted as fold difference compared to the respective H_2_O_2_ treated control. (c) Lipid peroxidation was assessed by measuring MDA levels by TBARS method. Cells were treated with 1 mM H_2_O_2_ for 6 h with or without treatment with the indicated concentrations of SAC. MDA levels are represented as fold change over control. *∗∗*P<0.01 and *∗∗∗*P<0.001 compared to the control group. #P<0.05, ##P<0.01, and ###P<0.001 compared to the H_2_O_2_ treated group.

ROS production is by far one of the triggering factors for apoptosis induction [25]. Since SAC is a potent ROS scavenger [8], we thought of investigating whether SAC inhibits apoptotic cell death via scavenging ROS in H_2_O_2_ treated HepG2 cells. First we performed a time course experiment with 1 mM H_2_O_2_ and found that 2 h time point showed maximal ROS levels (data not shown). To investigate the effect of SAC on cellular ROS levels, cells were treated with SAC in the presence or absence of H_2_O_2_ and ROS accumulation was measured using the well characterized DCFH_2_-DA fluorescence assay followed by FACS analysis. We would like to mention that the added H_2_O_2_ that enters cells and H_2_O_2_ dependent further ROS production in cells, contribute to the increase in cellular ROS levels. According to the result (Figure 3(a)), the intensity of DCF fluorescence, which is indicative of the ROS levels, was significantly elevated by 1.7 fold after treatment with 1 mM H_2_O_2_ from the ROS levels of the untreated control sample which was set at 1. However, SAC treatment significantly reduced the ROS levels (1.2 fold for 100 *μ*M SAC and 1 fold for 150 *μ*M SAC) in a dose dependent manner. Furthermore, in cells treated with 150 *μ*M SAC alone there was no significant change in ROS levels over control.

To avoid the possibility of SAC scavenging added H_2_O_2_ and causing the results in Figure 3(a), we first treated cells with H_2_O_2_ for 30 min, washed it off with phosphate buffered saline and then followed by SAC treatment for the indicated time points (Figure 3(b)). SAC was able to significantly inhibit H_2_O_2_ induced ROS accumulation in cells when treated for the indicated time points post H_2_O_2_ treatment as evident from the fold change in DCF fluorescence of cells treated with both H_2_O_2_ and SAC compared to their respective H_2_O_2_ treated controls (Figure 3(b)). This experiment clearly demonstrates that SAC is able to scavenge the H_2_O_2_ induced ROS accumulation in cells, a hallmark of oxidative stress in cells.

### 3.6. SAC Inhibits H_2_O_2_ Induced Lipid Peroxidation in HepG2 Cells

Excessive ROS levels leads to lipid peroxidation which is indicative of cellular oxidative stress. Lipid peroxidation was estimated via measuring the levels of malondialdehyde (MDA) in cells [17]. The result shows that treatment of HepG2 cells with H_2_O_2_ leads to significant increase in MDA levels as a result of increased levels of lipid peroxidation in comparison to the control. However, pretreatment with SAC decreases MDA levels (Figure 3(c)). SAC alone sample showed no significant effect over control on lipid peroxidation in cells.

### 3.7. SAC Up-Regulates HO-1 in H_2_O_2_ Treated HepG2 Cells by Increasing Nuclear Accumulation of Nrf-2 via Akt Phosphorylation

**Figure 4:**
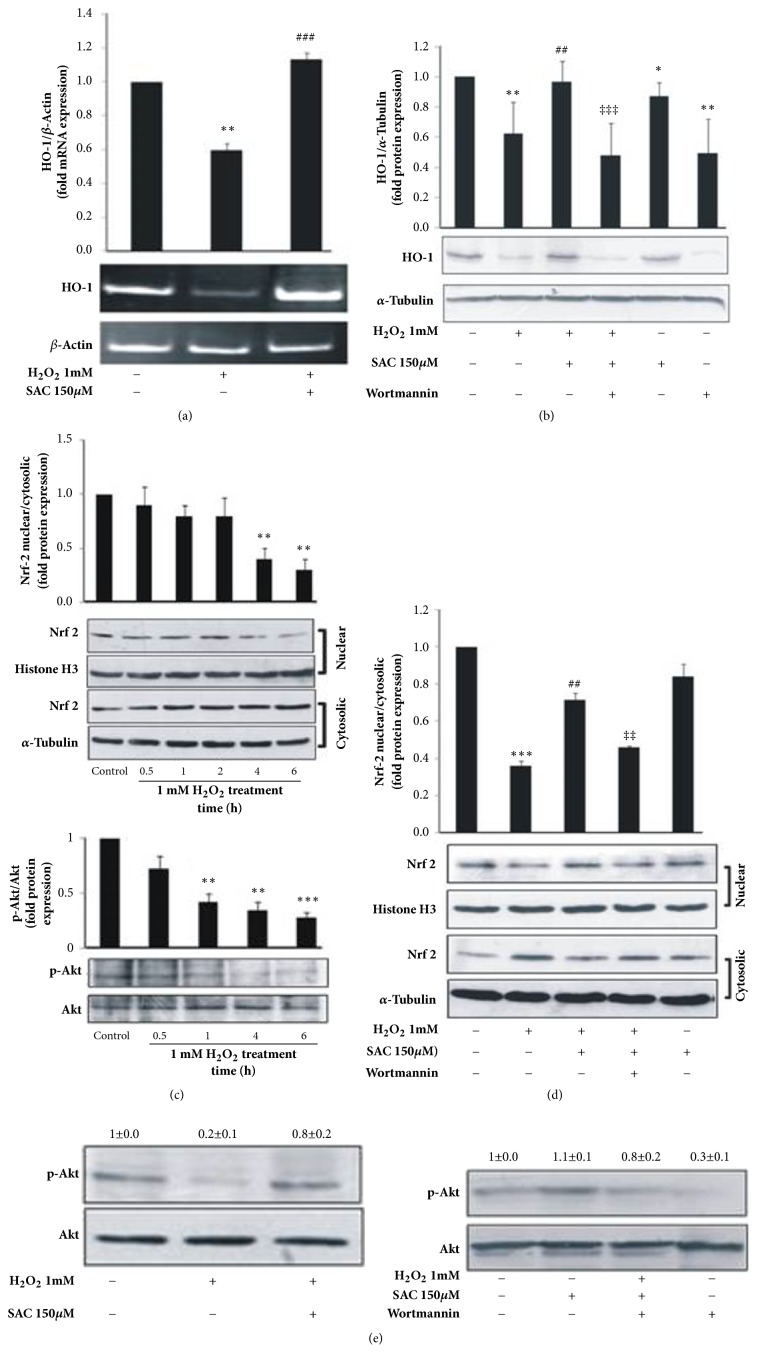
SAC activates the Nrf-2/HO-1 pathway via the induction of Akt phosphorylation in H_2_O_2_ induced HepG2 cells. (a) Cells were treated with 1 mM H_2_O_2_ for 24 h with or without treatment with SAC. PCR was performed to determine HO-1 mRNA levels. A representative PCR gel is shown. The HO-1/*β*-actin ratio was determined using densitometry and the fold mRNA expression is represented as a bar graph. Densitometry data represents mean ± SD of four independent experiments. (b) Cells were treated with 1 mM H_2_O_2_ for 24 h with or without treatment with SAC in the presence or absence of 10 *μ*M wortmannin. Western blot with whole cell lysate was performed to determine HO-1 protein levels. *α*-Tubulin was used as an internal loading control. The HO-1/*α*-Tubulin ratio was determined using densitometry and the fold protein expression is represented as a bar graph. Densitometry data represents mean ± SD of three independent experiments and a representative blot is shown below. (c) Cells were treated with 1 mM H_2_O_2_ for the indicated time points. Upper panel: Nrf-2 protein levels in both nuclear and cytosolic extracts were determined using western blot analysis. A representative western blot gel is shown. The ratio of the band intensities of nuclear (normalized to that of nuclear Histone H3) and cytosolic (normalized to that of cytosolic *α*-tubulin) levels of Nrf-2 was determined using densitometry and the fold protein expression is represented as a bar graph. Densitometry data represents mean ± SD of three independent experiments. Lower panel: Western blot with whole cell lysate was used to determine Akt phosphorylation levels using p-Akt antibody and the blot was reblotted for Akt levels (loading control). The band intensities of p-Akt levels were normalized to that of Akt and the data represented as mean ± SD of three independent experiments above a representative western blot. (d) Cells were treated with 1 mM H_2_O_2_ for 6 h with or without treatment with SAC in the presence or absence of 10 *μ*M wortmannin. Nrf-2 protein levels in both nuclear and cytosolic extracts were determined using western blot analysis. A representative western blot gel is shown. The ratio of the band intensities of nuclear (normalized to that of nuclear Histone H3) and cytosolic (normalized to that of cytosolic *α*-tubulin) levels of Nrf-2 was determined using densitometry and the fold protein expression is represented as a bar graph. Densitometry data represents mean ± SD of three independent experiments. (e) Cells were treated with 1 mM H_2_O_2_ for 6 h with or without treatment with SAC in the presence or absence of 10 *μ*M wortmannin. Western blot with whole cell lysate was used to determine Akt phosphorylation levels using p-Akt antibody and the blot was reblotted for Akt levels (loading control). The band intensities of p-Akt levels were normalized to that of Akt and the data represented as mean ± SD of three independent experiments above a representative western blot. *∗*P<0.05 and *∗∗*P<0.01 *∗∗∗*P<0.001 compared to the control group. ##P<0.01, ###P<0.001 compared to the H_2_O_2_ treated group. ‡‡P<0.01 and ‡‡‡P<0.001 compared to H_2_O_2_ and SAC treated group.

Heme oxygenase-1 (HO-1) is a key endogenous antioxidant enzyme which has been reported to protect cells from oxidative stress induced apoptosis [5, 6] by regulating cellular ROS levels. Thus to elucidate the mechanism underlying SAC's hepatoprotective role against H_2_O_2_ induced oxidative injury in HepG2 cells, we assessed the effect of SAC on HO-1 levels. We find that, H_2_O_2_ treatment decreased the mRNA levels of HO-1 and SAC treatment increased these levels (Figure 4(a)). At the protein level, H_2_O_2_ treatment down regulated HO-1 and again SAC treatment increased these levels. Treatment with SAC alone has no significant effect on endogenous HO-1 levels (Figure 4(b)).

To elucidate how SAC upregulated HO-1, we assessed the effect of SAC on the nuclear translocation of Nrf-2, the transcriptional regulator of HO-1. Our time course experiment using 1 mM H_2_O_2_ revealed that nuclear levels of Nrf-2 decreased with increase in time of H_2_O_2_ treatment, with 6 h time point showing maximal reduction in nuclear levels of Nrf-2 over control (Figure 4(c), upper panel) as a consequence of delayed response to oxidative stress [26, 27]. Thus we chose this time point for our experiments. According to Figure 4(d), H_2_O_2_ treatment decreased (0.36 ± 0.03) the nuclear levels of Nrf-2 over control (1) and SAC treatment increased Nrf-2 levels in the nuclear extract in H_2_O_2_ treated HepG2 cells (0.71 ± 0.04) as compared to the H_2_O_2_ treated cells in the absence of SAC (Figure 4(d)). Histone H3 was used as an internal control for nuclear extract and *α*-tubulin was used as an internal control for cytosolic extract.

Afterwards, we investigated the effects of SAC on Akt phosphorylation, which plays an important role in protecting cells from oxidative injury by modulating the Nrf-2/HO-1 pathway [28–30]. We found that Akt phosphorylation was inhibited by 1 mM H_2_O_2_ treatment in a time dependent manner with significant inhibition at 6 hours (Figure 4(c), lower panel). Our result showed that Akt phosphorylation that was inhibited by H_2_O_2_ treatment was induced on treatment with SAC. Treatment with SAC alone showed no significant effect on phosphorylated Akt levels from that of control (Figure 4(e)).

To confirm the involvement of SAC mediated Akt phosphorylation on the upregulation of HO-1 protein expression, cells were treated with H_2_O_2_ and SAC in the presence of the PI3K inhibitor wortmannin. Akt phosphorylation was inhibited when cells were treated with wortmannin (Figure 4(e), right panel, lane 4). Also, SAC failed to induce Akt phosphorylation in cells treated with wortmannin in the presence of H_2_O_2_ (Figure 4(e), right panel, lane 3). Likewise, Figure 4(b) (lane 4) clearly demonstrates that SAC was unable to induce HO-1 expression in cells when Akt phosphorylation was inhibited by wortmannin. From Figure 4(b) it is evident that wortmannin treatment decreased HO-1 protein levels below control. We chose the concentration of wortmannin as 10 *μ*M for our treatments with the intention of inhibiting the basal levels of HO-1 induction in control cells which ensues in these cells due to basal levels of Akt phosphorylation as evident from Figure 4(e) (see controls). Comparison of lane 1 and lane 6 of Figure 4(b) which are without and with wortmannin respectively points out that HO-1 expression in HepG2 cells is regulated via the Akt signaling pathway. Additionally, Figure 4(d) (lane 4) demonstrates that, SAC failed to increase the nuclear levels of Nrf-2 in the presence of wortmannin and H_2_O_2_ compared to the SAC and H_2_O_2_ treated group in the absence of wortmannin (Figure 4(d), lane** 3**). These results clearly suggest that SAC upregulates HO-1 via activating the Akt /Nrf-2 pathways.

### 3.8. SAC Inhibits Apoptosis in H_2_O_2_ Stimulated HepG2 Cells by Upregulating HO-1

**Figure 5:**
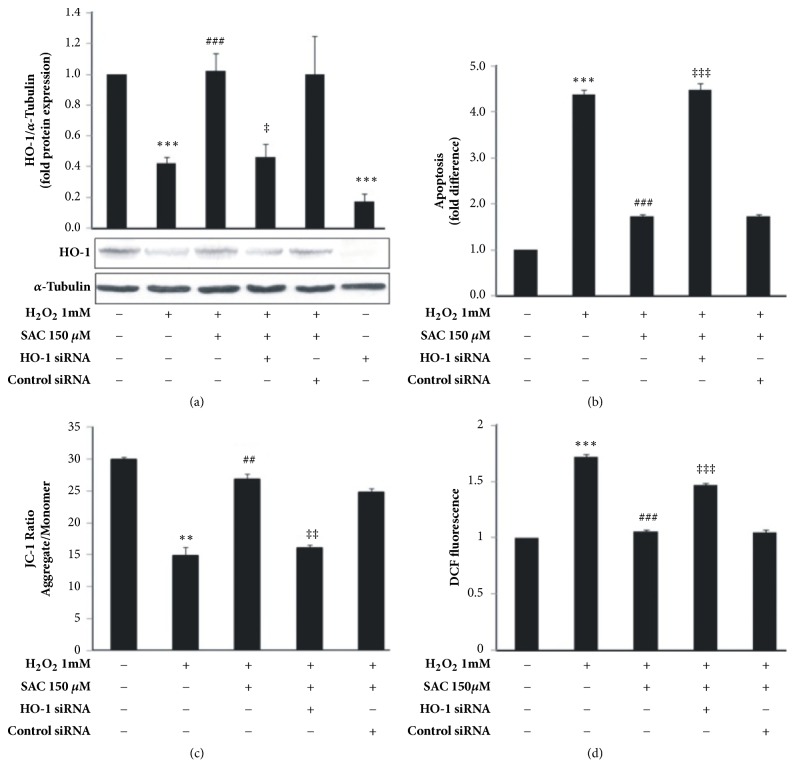
SAC inhibits H_2_O_2_ induced apoptosis in HepG2 cells via upregulation of HO-1. Cells were transfected with HO-1 specific siRNA or control siRNA and incubated for 72 h followed by treatment with H_2_O_2_ for 24 h in the presence or absence of SAC. (a) Western blot with whole cell lysate was used to determine HO-1 protein levels. *α*-Tubulin was used as a loading control. The HO-1/*α*-Tubulin ratio was determined using densitometry and the fold protein expression is represented as a bar graph. Densitometry data represents mean ± SD of three independent experiments and a representative western blot gel image is shown below. (b) Cells were stained with Annexin V/PI followed by FACS analysis. Fold difference of apoptosis compared to control was plotted. (c) Mitochondrial membrane potential (∆Ψm) was estimated by JC-1 staining followed by FACS analysis. The ratio of aggregate and monomeric form of JC-1 was plotted. (d) HO-1 specific siRNA or control siRNA transfected cells were treated with H_2_O_2_ for 2 h with or without treatment with SAC. ROS level was measured by DCFH_2_-DA staining followed by FACS analysis and represented as DCF fluorescence. Fluorescence intensities were plotted as a bar graph. *∗∗*P<0.01 and *∗∗∗*P<0.001 compared to the control group. ##P<0.01 and ###P<0.001 compared to the H_2_O_2_ treated group. ‡P<0.05, ‡‡P<0.01, and ‡‡‡P<0.001 compared to the control siRNA treated group. All data are represented by mean ± SD of three independent experiments.

To directly confirm the involvement of HO-1 in SAC mediated apoptosis inhibition, silencing of HO-1 gene using HO-1 specific siRNA was performed. Transfecting cells with HO-1 specific siRNA significantly reduced HO-1 levels compared to control (Figure 5(a) compare lanes 1 and 6). Additionally we found that when HO-1 specific siRNA transfected cells were treated with SAC and H_2_O_2_, SAC failed to upregulate HO-1 levels compared to that in control siRNA transfected cells (Figure 5(a), lanes 4 and 5) in contrast to that in untransfected cells treated with SAC and H_2_O_2_ (Figure 5(a), lane 3).

Next, cells transfected with HO-1 siRNA or control siRNA were treated with SAC and H_2_O_2_ and subjected to Annexin V and PI staining followed by flow cytometry analysis. As depicted from Figure 5(b), H_2_O_2_ treatment increased apoptosis by 4.4 ± 0.12 fold over control which was considered as 1. SAC treatment decreased apoptosis of H_2_O_2_ treated cells by 1.7 ± 0.05 fold. Interestingly, SAC failed to inhibit H_2_O_2_ induced apoptosis in HO-1 knocked down cells when compared to the control siRNA transfected cells treated with SAC and H_2_O_2_ (compare lanes 4 and 5, fold apoptosis 4.5 ± 0.14 and 1.7 ± 0.04, respectively in Figure 5(b)). This result directly indicates that SAC mediated upregulation of HO-1 is responsible for protecting HepG2 cells from H_2_O_2_ induced apoptosis.

The involvement of HO-1 induction in regulating mitochondrial membrane potential was demonstrated when HepG2 cells transfected with HO-1 siRNA or control siRNA were treated with SAC and H_2_O_2_ and subjected to JC-1 staining. As depicted in Figure 5(c) mitochondrial membrane potential increased after SAC treatment (27 ± 0.9) as compared to H_2_O_2_ treated cells (15 ± 1.3). However, treatment with SAC failed to restore ∆Ψ_m_ in H_2_O_2_ and HO-1 siRNA transfected group in comparison to the control siRNA transfected cells also treated with SAC and H_2_O_2_ (compare lanes 4 and 5 in Figure 5(c)). This result suggests that SAC induced restoration of H_2_O_2_ mediated loss of ∆Ψ_m_ is mediated by increased expression of the antioxidant enzyme HO-1.

### 3.9. SAC Inhibits ROS Accumulation in H_2_O_2_ Induced HepG2 Cells via Up-Regulation of HO-1

To investigate whether the upregulation of HO-1 by SAC is one of the mechanisms by which it decreases ROS levels in cells, HepG2 cells were transfected with HO-1 siRNA before treatment with SAC and H_2_O_2_ and then subjected to DCFH_2_-DA FACS assay. As indicated in Figure 5(d), in untransfected cells treatment with 1 mM H_2_O_2_ elevated ROS levels by 1.7 fold compared to the control. SAC treatment significantly reduced H_2_O_2_ induced ROS levels to control levels. However, SAC failed to inhibit H_2_O_2_ stimulated ROS levels in HO-1 knocked down cells (Figure 5(d), lane 4) compared to control siRNA transfected cells (Figure 5(d), lane 5), suggesting that SAC decreases cellular ROS levels via upregulation of HO-1.

## 4. Discussion

In this work we demonstrated the molecular mechanism by which SAC renders antioxidative and antiapoptotic potential against H_2_O_2_ induced cytotoxicity in HepG2 cells. We first determined the cytoprotective effects of SAC in H_2_O_2_ treated HepG2 cells. Our results demonstrated that treatment with H_2_O_2_ significantly inhibited HepG2 cell viability in a concentration and time dependent manner (Figures 1(a) and 1(b)). SAC pretreatment was found to dose dependently increase cell viability in H_2_O_2_ treated HepG2 cells (Figure 1(c)). When cells were first treated with H_2_O_2_ followed by SAC treatment (posttreatment), SAC was found to decrease H_2_O_2_ induced cytotoxicity at different time points (Figure 1(d)). Interestingly, SAC treatment evoked an increase in cytoprotection compared to control with an increase in time of treatment as well as an increase in cytotoxicity, thus confirming the cytoprotective role of SAC in H_2_O_2_ treated HepG2 cells. However, higher concentration of SAC (500 *μ*M) decreased cell viability when added with H_2_O_2_ (Figure 1(c)) consistent with the fact that low amount of toxicity was observed with 500 *μ*M SAC treatment alone in HepG2 cells (Figure 1(e)). Since 500 *μ*M SAC treatment induced some amount of toxicity in cells, it was inefficient in reversing the toxic effect of H_2_O_2_ compared to lower doses of SAC.

ROS production is an important marker of oxidative stress. SAC has been reported to directly scavenge hydroxyl radical, a byproduct of H_2_O_2_ decomposition [31]. In our study we also found that SAC inhibited H_2_O_2_ induced ROS accumulation in cells (which includes the added H_2_O_2_ that enters cells and the H_2_O_2_ induced further ROS production) both when added prior to and post-H_2_O_2_ treatment (Figures 3(a) and 3(b)). H_2_O_2_ induced ROS production leads to lipid peroxidation, another hallmark of oxidative stress. In our study we found that SAC was able to significantly inhibit H_2_O_2_ induced lipid peroxidation (Figure 3(c)) in cells. Since our lipid peroxidation experiment which was performed after 6 h of H_2_O_2_ treatment, shows an increase in lipid peroxidation by 3.5-fold which would be possible only via further induced intracellular ROS production (hydroxyl radical and the peroxyl radical cause lipid peroxidation), and since SAC reduced DCF fluorescence post 6 h of H_2_O_2_ treatment (Figure 3(b)), we could speculate that SAC scavenges both the added ROS that enters cells and the intracellularly produced ROS in HepG2 cells. Thus we could suggest that the ROS scavenging activity SAC in H_2_O_2_ treated HepG2 cells could be one of the mechanisms by which SAC protects cells from oxidative injury.

H_2_O_2_ induced ROS production eventually leads to apoptotic cell death in hepatocytes [20].  Figure 2(a) suggests that SAC inhibited H_2_O_2_ mediated increase in the sub-G1 peak produced as a result of DNA fragmentation which is an indication of cell death. From Annexin V and PI double staining assay we found that SAC decreased the number of Annexin V positive cells significantly (Figure 2(b)) suggesting that SAC indeed inhibits apoptosis in HepG2 cells. The above results clearly elucidate that SAC exerts antioxidative and antiapoptotic effects in H_2_O_2_ induced HepG2 cells.

We evaluated the effect of SAC on the hallmarks of apoptosis such as disruption of mitochondrial membrane potential [32], release of mitochondrial cytochrome c, alteration in the Bax/Bcl-2 ratio, and proteolytic cleavage of caspase-3. We found that SAC inhibited H_2_O_2_ mediated loss of mitochondrial membrane potential (Figure 2(f)), prevented mitochondrial cytochrome c release (Figure 2(d)), decreased the Bax/Bcl-2 ratio (Figure 2(c)), and inhibited caspase-3 cleavage (Figure 2(e)). Thus our results clearly demonstrate that SAC inhibits H_2_O_2_ induced apoptosis through modulation of the Bcl-2/Bax dependent cell death pathways.

Next we wanted to determine the molecular mechanism behind the antiapoptotic and antioxidative role of SAC. Various animal studies suggest that SAC protects from oxidative injury via modulation of anti-oxidant enzymes [33–35]. SAC was found to protect hepatocytes from chromium induced hepatotoxicity by decreasing ROS and increasing antioxidant levels [13]. Previous reports demonstrate that upregulation of endogenous HO-1 by cobalt protoporphyrin (CoPP) rescued mice from immune-mediated apoptotic liver damage and prolonged survival [36]. HO-1 induction also protected isolated primary hepatocytes from anti-CD95-induced apoptosis* in vitro *[36]. Ben-Ari et al. 2013, showed that induction of HO-1 by CoPP protects the liver from apoptosis induced I/R injury. HO-1 induction revealed fewer apoptotic hepatocytes cells, lesser caspase-3 levels, and decreased mean number of proliferating cells (positively stained for Ki67) [6]. Thus, we wanted to determine whether HO-1 was a molecular target of SAC. We first determined whether SAC regulated the expression of HO-1 in HepG2 cells as a protective mechanism against H_2_O_2_ induced oxidative injury and apoptosis. For this we assessed the levels of the antioxidant enzyme HO-1 in the presence and absence of SAC. We found that treatment with SAC augmented both the mRNA and protein levels of HO-1 in H_2_O_2_ treated HepG2 cells (Figures 4(a) and 4(b)).

In order to identify the mechanism by which SAC upregulates HO-1 expression, we assessed the effects of SAC on the nuclear translocation of Nrf-2, the transcription factor that regulates the expression of HO-1. Nuclear accumulation of Nrf-2 depends on the dose and time of H_2_O_2_ exposure to cells. It has been reported that low and moderate (0.125 mmol/L, 0.25 mmol/L, and 0.5 mmol/L) doses of H_2_O_2_ exposure of rat pulmonary microvascular endothelial cells led to the nuclear accumulation of Nrf-2, increased transcriptional activity, and induction of ARE-medicated gene expression. In contrast, exposure of cells to high doses of H_2_O_2_ (1 mmol/L, 2 mmol/L) led to the nuclear exclusion of Nrf-2, decreased transcriptional activity and downregulation of ARE-mediated gene expression [26]. Jain et al. reported that H_2_O_2_ induced Nrf-2 nuclear localization occurs in a time dependent manner in HepG2 cells. After 1 h of 4 mM H_2_O_2_ treatment, Nrf-2 translocates to the nucleus as an early response to oxidative stress. However, Nrf-2 was exported out of the nucleus at 4 h after 4 mM H_2_O_2_ treatment as a result of delayed response to oxidative stress [27]. For our experiments we used 1 mM H_2_O_2_ and treated cells for 6 h, which is a dose and time that exceeds cellular defence mechanisms and mimics a state of cellular oxidative injury and at this dose we have found that Nrf-2 nuclear levels are decreased over control (Figure 4(c), upper panel). Additionally, SAC treatment increased the nuclear translocation of Nrf-2, as evidenced by the comparison of the nuclear levels of Nrf-2 in the H_2_O_2_ treated cells in the presence and absence of SAC (Figure 4(d)).

Since the PI3K-Akt signaling pathway has been reported to play important roles in preventing oxidative stress in cells by suppressing apoptosis and promoting cell growth and proliferation via upregulation of HO-1 expression [37–39] in an Nrf-2 dependent manner [28–30], we sought to investigate whether SAC had any effects on the phosphorylation of Akt. In our study, treatment of cells with 1mM concentration of H_2_O_2_ mimics high ROS levels that induce apoptosis. Under this scenario we find that Akt phosphorylation is inhibited (Figure 4(e), left panel) which correlates with an induction of apoptosis. Our data is in support of published literature which states that ROS induces apoptosis by inhibiting Akt phosphorylation [30, 40]. Our results show that Akt phosphorylation increased with SAC treatment (Figure 4(e), left panel). We also find that SAC was unable to increase Akt phosphorylation in the presence of the Akt inhibitor wortmannin (Figure 4(e), right panel) which specifically inhibits PI3K, suggesting that SAC probably acts at the level or upstream of PI3K in the pathway. To directly elucidate the involvement of the Akt/Nrf-2 pathway in SAC mediated upregulation of HO-1 expression, HO-1 levels were determined in the presence of wortmannin. SAC was unable to induce HO-1 expression in cells when Akt phosphorylation was inhibited by wortmannin (Figure 4(b)). Also SAC failed to induce the nuclear translocation of Nrf-2 in the presence of wortmannin (Figure 4(d)). Thus we could conclude that SAC upregulated HO-1 in an Akt/Nrf-2 dependent manner. In our SAC alone treated control, we found that SAC failed to increase Akt phosphorylation (Figure 4(e) right panel) and likewise HO-1 expression (Figure 4(b)) over control levels. Since our result demonstrates that SAC regulates HO-1 via Akt phosphorylation only under H_2_O_2_ induction, we could speculate that HO-1 induction could be one of the mechanisms via which SAC renders cytoprotection to cells under oxidative stress.

Finally, to corroborate the involvement of SAC mediated upregulation of HO-1 enzyme in protecting HepG2 cells from H_2_O_2_ induced apoptosis, we knocked down HO-1 gene using HO-1 specific siRNA. As depicted from Figure 5(b), SAC failed to inhibit H_2_O_2_ induced apoptosis in HO-1 knocked down cells as compared to control siRNA transfected cells (Figure 5(b)). Additionally in HO-1 knocked down cells, SAC failed to restore the loss of ∆Ψ_m_ in H_2_O_2_ treated cells (Figure 5(c), lane 4). This result directly indicates that SAC mediated upregulation of HO-1 is responsible for protecting HepG2 cells from H_2_O_2_ induced apoptosis.

Now to investigate whether SAC mediated upregulation of HO-1 is one of the mechanisms by which it decreases ROS levels in cells, we assessed ROS levels in HO-1 knocked down cells in the presence of H_2_O_2_ and SAC. We found that ROS levels in HO-1 siRNA transfected cells treated with both H_2_O_2_ and SAC (Figure 5(d), lane 4) were higher than the control siRNA transfected cells treated with H_2_O_2_ and SAC (Figure 5(d), lane 5). This result points to the involvement of HO-1 in SAC mediated decrease in ROS levels and specifically SAC decreases cellular ROS levels via upregulation of HO-1.

## 5. Conclusion

In summary SAC exerts cytoprotective effects reversing H_2_O_2_ induced oxidative damage in HepG2 cells. SAC prevents H_2_O_2_ induced ROS production, lipid peroxidation, and cellular apoptosis. For the first time we demonstrate that the ROS scavenging activity of SAC via upregulating the antioxidant enzyme HO-1 in an Akt/ Nrf-2 dependent manner is a crucial mechanism underlying the antiapoptotic and antioxidative potential of SAC in HepG2 cells. This work provides valuable insights into the hepatoprotective mechanisms of SAC and portrays a promising therapeutic role of this garlic compound in combating liver oxidative stress and apoptosis which underlie a majority of liver diseases.

## Data Availability

The data used to support the findings of this study are included within the article.
